# Transcript Expression Profiles and MicroRNA Regulation Indicate an Upregulation of Processes Linked to Oxidative Stress, DNA Repair, Cell Death, and Inflammation in Type 1 Diabetes Mellitus Patients

**DOI:** 10.1155/2022/3511329

**Published:** 2022-02-01

**Authors:** Paula Takahashi, Danilo J. Xavier, Jessica E. B. F. Lima, Adriane F. Evangelista, Cristhianna V. A. Collares, Maria C. Foss-Freitas, Diane M. Rassi, Eduardo A. Donadi, Geraldo A. Passos, Elza T. Sakamoto-Hojo

**Affiliations:** ^1^Department of Genetics, Ribeirão Preto Medical School, University of São Paulo (USP), Ribeirão Preto, 14049900, SP, Brazil; ^2^Molecular Oncology Research Center, Barretos Cancer Hospital, Barretos, SP, Brazil; ^3^Division of Clinical Immunology, Ribeirão Preto Medical School, University of São Paulo (USP), Ribeirão Preto, SP, Brazil; ^4^Division of Endocrinology, Department of Internal Medicine, Ribeirão Preto Medical School, University of São Paulo (USP), Ribeirão Preto, SP, Brazil; ^5^Department of Pharmacology, Ribeirão Preto Medical School, University of São Paulo (USP), Ribeirão Preto, SP, Brazil; ^6^Laboratory of Genetics and Molecular Biology, Department of Basic and Oral Biology, School of Dentistry of Ribeirão Preto, University of São Paulo (USP), Ribeirão Preto, SP, Brazil; ^7^Department of Biology, Faculty of Philosophy, Sciences and Letters of Ribeirão Preto, University of São Paulo (USP), Ribeirão Preto, SP, Brazil

## Abstract

Type 1 diabetes (T1D) arises from autoimmune-mediated destruction of insulin-producing *β*-cells leading to impaired insulin secretion and hyperglycemia. T1D is accompanied by DNA damage, oxidative stress, and inflammation, although there is still scarce information about the oxidative stress response and DNA repair in T1D pathogenesis. We used the microarray method to assess mRNA expression profiles in peripheral blood mononuclear cells (PBMCs) of 19 T1D patients compared to 11 controls and identify mRNA targets of microRNAs that were previously reported for T1D patients. We found 277 differentially expressed genes (220 upregulated and 57 downregulated) in T1D patients compared to controls. Analysis by gene sets (GSA and GSEA) showed an upregulation of processes linked to ROS generation, oxidative stress, inflammation, cell death, ER stress, and DNA repair in T1D patients. Besides, genes related to oxidative stress responses and DNA repair (*PTGS2*, *ATF3*, *FOSB*, *DUSP1*, and *TNFAIP3*) were found to be targets of four microRNAs (hsa-miR-101, hsa-miR148a, hsa-miR-27b, and hsa-miR-424). The expression levels of these mRNAs and microRNAs were confirmed by qRT-PCR. Therefore, the present study on differential expression profiles indicates relevant biological functions related to oxidative stress response, DNA repair, inflammation, and apoptosis in PBMCs of T1D patients relative to controls. We also report new insights regarding microRNA-mRNA interactions, which may play important roles in the T1D pathogenesis.

## 1. Introduction

Type 1 diabetes (T1D) or insulin-dependent diabetes mellitus (IDDM) is a polygenic disorder possibly triggered by environmental factors that results from a T cell-mediated autoimmune attack against the insulin-producing *β*-cells localized in the pancreatic islets of Langerhans [1]. The typical pathological lesion is a destructive immune cell infiltrate (insulitis), affecting insulin-producing *β*-cells at several levels and impairing insulin synthesis as a consequence [2]. During this process, islet-infiltrating mononuclear cells release proinflammatory cytokines and specific biochemical markers in the serum of patients, which have been exploited as potential markers for the pathogenesis of T1D. In fact, the levels of inflammatory markers have been significantly upregulated in T1D patients compared to healthy human subjects [3].

The hallmark of T1D is a decreased insulin secretion that is subsequently succeeded by chronic hyperglycemia, which has been implicated in long-term complications affecting several organs, including the kidneys, eyes, heart, nerves, and blood vessels [4]. Chronic hyperglycemia induces the production of reactive oxygen species (ROS), which in excess can overwhelm the antioxidant system and lead to oxidative stress [5, 6]. There is evidence that oxidative stress can increase the release of proinflammatory cytokines, subsequently leading to inflammation and *β*-cell destruction, contributing to T1D progression [7]. Nevertheless, studies have reported that T1D patients present elevated oxidative stress markers [8–10] and decreased antioxidant capacity [7, 9, 11]. Besides, T1D patients have shown higher levels of DNA damage and oxidative DNA damage than controls [12, 13], indicating that DNA repair mechanisms may be compromised in those patients. However, there is still scarce information about molecular signaling pathways and genes implicated in biological processes related to oxidative stress responses and DNA repair in T1D.

Moreover, there are reports that point out to mRNA-microRNA interactions that might be involved in T1D pathogenesis [14, 15]. In fact, since microRNAs act as gene expression regulators, they might play some roles in the pathogenesis of human diseases. The miR-21 increased *β*-cell apoptosis through degradation of mRNA *BCL2* transcript in mouse models of T1D and human cells [16]. By inhibiting GLP-1 expression, the miR-192 inhibits insulin secretion [17]. Other differentially expressed microRNAs have been implicated in *β*-cell dysfunction, apoptosis [18], insulin secretion impairment [19], and inflammatory processes [20] through mRNA interactions. Some microRNAs are found consistently upregulated (miR-24-3p, miR-148a-3p, miR-181a-5p, miR-210-5p, and miR-375) or downregulated (miR-146a-5p, miR-150-5p, miR-342-3p, miR-1275, and miR-100-5p) in T1D patients compared to nondiabetic controls [14]. Still, there is much to clarify about the role of microRNAs in T1D development.

In the present study, we applied the microarray method to study the mRNA transcript expression profiles displayed by peripheral blood mononuclear cells (PBMCs) of T1D patients compared to healthy subjects. We also aimed to identify potential mRNAs targets that are mainly associated with responses to oxidative stress and DNA repair pathways and those associated with differentially expressed microRNAs (that were previously reported by Takahashi et al. [21]).

## 2. Material and Methods

### 2.1. Study Subjects

A total of 19 patients with type 1 diabetes (7 women and 12 men, with age ranging from 18 to 37), recruited while undergoing regular follow-up at the Outpatient Endocrinology of the Clinical Hospital of the Faculty of Medicine of Ribeirão Preto (HC/FMRP-USP), Brazil, and 11 healthy subjects (control group) (6 women and 5 men, with age ranging from 20 to 31) participated in the present study. The main characteristics of all participants are described in Tables 1 and 2. All patients were receiving treatment with human insulin, and those presenting recent episodes of ketoacidosis and late diabetic complications were excluded from the study. The study was conducted according to the guidelines of the Declaration of Helsinki and approved by the Local Ethics Committee of Clinical Hospital–Ribeirão Preto Medical School, University of São Paulo (protocol code no. 13314/2011 in November 23, 2011). Informed written consent was obtained from all participants.

### 2.2. Sample Collection, Isolation of Peripheral Blood Mononuclear Cells (PBMCs), and RNA Extraction

Peripheral blood samples (20 mL) were collected from all participants, followed by isolation of PBMCs using Histopaque-1077 (Sigma-Aldrich, Inc., USA). Total RNA was extracted using Trizol reagent (Invitrogen, Life Technologies, Carlsbad, CA, USA) according to manufacturer's instructions. The quality and quantification of RNA samples were measured using the NanoDrop ND-1000 Spectrophotometer (Uniscience, São Paulo, Brazil). The integrity of RNA samples was evaluated using Agilent RNA Nano 6000 chips onto the Agilent 2100 Bioanalyzer (Agilent Technologies, Santa Clara, CA, USA). RNA samples that were protein- and phenol-free, and RNA integrity number (RIN) ≥ 7.0 were considered for the microarray and qRT-PCR analysis.

### 2.3. mRNA/miRNA Microarrays and qRT-PCR

The microarray technique and the expression data normalization and statistical analysis were performed as previously described [21, 22]. The microarray data from all samples used in this study are publicly available in the ArrayExpress database (http://www.ebi.ac.uk/arrayexpress) under the accession numbers E-MEXP-3348 (T1D group) and E-MEXP-3963 (control group). The qRT-PCR method was used to validate the expression results obtained for mRNA and microRNA microarrays. For microRNA expression level analysis, 10 ng of total RNA was reverse transcribed using Taqman microRNA Reverse Transcription kit (Applied Biosystems, Foster City, CA). The qRT-PCR was performed using Taqman Universal PCR Master Mix with AmpErase uracil N-glycosylase (UNG) (Applied Biosystems) and Taqman MicroRNA Assays (Applied Biosystems) for the following microRNAs: hsa-miR-101 (MIMAT0000099) (002253), hsa-miR-148a (MIMAT0000243) (000470), hsa-miR-27b (MIMAT0000419) (000409), hsa-miR-424 (MIMAT0001341) (000604), and RNU48 (001006). For mRNA expression levels, one *μ*g of total RNA was reverse transcribed using Superscript III First-Strand Synthesis System (Invitrogen) after DNAse (Invitrogen) treatment. The qRT-PCR was performed using Taqman Universal PCR Master Mix with AmpErase uracil N-glycosylase (UNG) (Applied Biosystems) and Taqman Gene Expression Assays (Applied Biosystems) for *ATF3* (Hs00231069_m1), *TNFAIP3* (Hs00234713_m1), *PTGS2* (Hs00153133_m1), *UCP3* (Hs01106052_m1), *DUSP1* (Hs00610256_g1), *FOSB* (Hs00171851_m1), *GAPDH* (Hs02758991_g1), and *HPRT1* (Hs02800695_m1). For both microRNA and mRNA, the reactions were carried out in triplicate in 96-well plates, sealed with MicroAmp® Optical Adhesive Film, and performed on a StepOnePlus Real-Time PCR System (Applied Biosystems). The microRNA and mRNA expression levels were obtained according to the 2^−ΔΔCt^ method [23]. To analyze the expression levels, both groups of patients and controls were subjected to the normality test D'Agostino-Pearson omnibus K2 followed by the Mann–Whitney. The *F*-test was also used to test the group variances, followed by the *T*-test for unpaired samples with Welch's correction. *P* values < 0.05 were considered statistically significant in the comparisons.

### 2.4. Gene Set Analysis (GSA) and Gene Set Enrichment Analysis (GSEA)

The analysis of gene sets was performed using BRB-ArrayTools (developed by Richard Simon and BRB-ArrayTools Development Team) to identify gene groups with significant expression (GSA) and significantly enriched gene sets (GSEA), according to Gene Ontology (GO) terms. The LS/KS permutation test and Efron-Tibshirani's GSA maxmean test with random 1,000 permutations were carried out with a threshold of 0.005 to filter statistically significant (GSA) and significantly enriched (GSEA) gene sets, respectively.

## 3. Results

After normalization and adjustment of data, the analysis performed by *rank products* revealed 277 differentially expressed genes (DEGs) in PBMCs of patients with T1D compared to controls (220 upregulated and 57 downregulated) (Table S1). Some of these genes have already been widely described as associated with T1D, including those related to inflammatory processes (Table 3).

**Figure 1:**
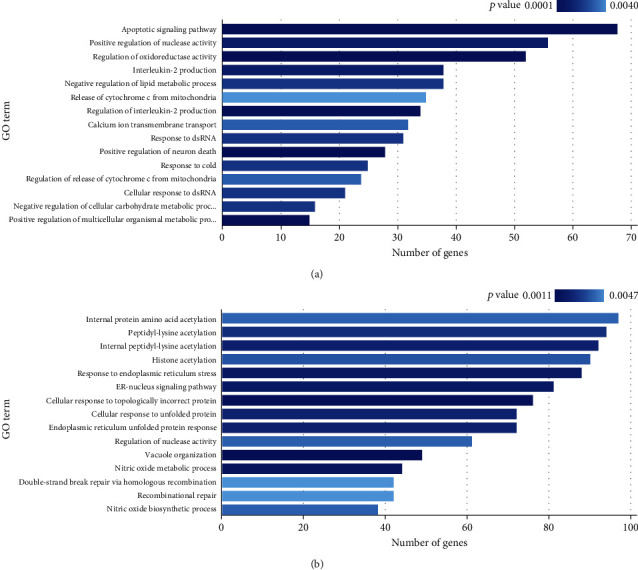
Biological processes that were found as differentially expressed/enriched in peripheral blood mononuclear cells (PBMCs) of T1D patients compared to the control group. (a) Gene set analysis (GSA) showing the first 15 biological processes related to significantly expressed genes in T1D patients compared to controls. (b) Gene set enrichment analysis (GSEA) showing the first 15 biological processes linked to significantly enriched genes in T1D patients compared to the controls. Only biological processes with *p* < 0.005 are shown.

In addition, the GSA and GSEA analysis indicated 49 significantly expressed gene sets (*p* < 0.005) and 55 statistically enriched gene sets (*p* < 0.005) in PBMCs of T1D patients compared to the control group. Biological processes related to apoptosis (apoptotic signaling pathway, release of cytochrome c from mitochondria), oxidative stress (regulation of oxidoreductase activity), inflammatory processes (interleukin-2 production), and cell death (positive regulation of neuron death) were found significantly upregulated in T1D patients. Pathways associated with endoplasmic reticulum stress and unfolded protein response (response to endoplasmic reticulum stress, cellular response to topologically incorrect protein/unfolded protein), reactive nitrogen species (nitric oxide metabolic and biosynthetic process), and DNA repair (double-strand break repair via homologous recombination and recombinational repair) were found statistically enriched for T1D patients (Figures 1(a) and 1(b)). The complete list of significant and statistically enriched gene sets can be found in Table S2.

Besides, to identify genes that were specifically related to the biological processes “response to oxidative stress” (GO: 0006979) and “DNA repair” (GO: 0006281), the list of DEGs was submitted to the gene prioritization tool Endeavor, which allowed the integration of the results in several gene databases. The 16 best ranked genes in each process and the respective fold-change values are highlighted (Table 4).

We also verified whether any of those 277 DEGs were included as possible targets for the 44 differentially expressed microRNAs (AUC ≥ 0.90), which were previously reported for the same group of patients [21] using the VENNY tool. We found 144 (52%) genes that may be potentially regulated by the differentially expressed microRNAs. Of these, 75 are possibly regulated by the upregulated microRNAs, seven by the downregulated microRNAs, and 62 by both up- and downregulated microRNAs (Table 5). Interestingly, among the possible targets, nine and thirteen genes belong to “response to oxidative stress” and “DNA repair” biological processes, respectively, such as *UCP3*, *PTGS2*, *ATF3*, *FOSB*, *DUSP1*, and *TNFAIP3* genes.

**Figure 2:**
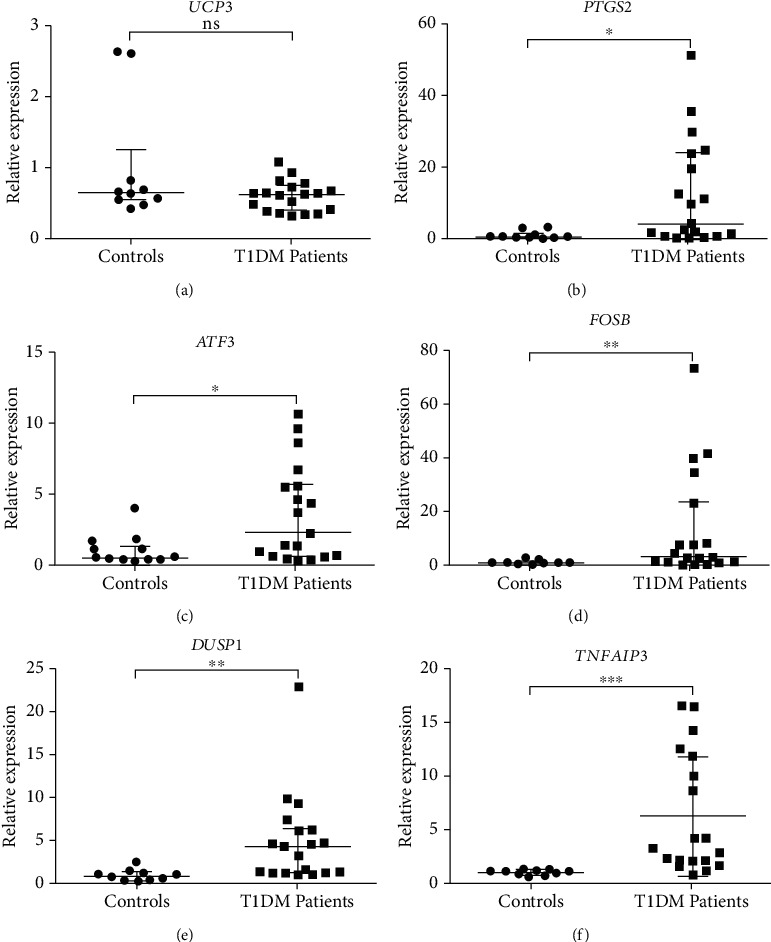
Relative expression of genes associated with oxidative stress response and DNA repair in PBMCs of T1D patients compared to the control group, evaluated by qRT-PCR. For all genes, the assay was performed for 19 T1D samples and 10 control samples. For *FOSB* (d), two controls were excluded (Co_01 and Co_11). These samples were the same used for the microarray method. Two endogenous genes were used to normalize the expression values: *GAPDH* and *HPRT1*. For the *UCP3* (a), *PTGS2* (b), *ATF3* (c), *FOSB* (d), and *DUSP1* (e), bars represent the median and interquartile range. For the *TNFAIP3* (f), bars represent the mean ± standard deviation. ∗ indicates statistically significant values for *p* < 0.05; ^∗∗^*p* < 0.01; ^∗∗∗^*p* < 0.001; ns: not significant.

**Figure 3:**
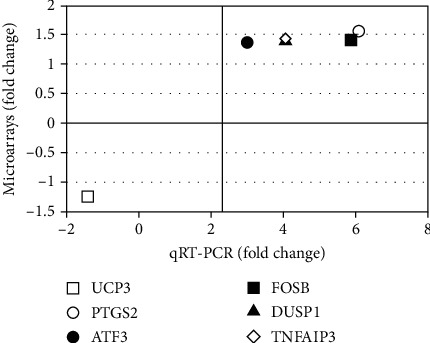
Comparison of fold change values (T1D versus control group), obtained by the microarrays and qRT-PCR methods, for the selected differentially expressed mRNAs.

To confirm the expression of some mRNAs, we selected six genes (*UCP3*, *PTGS2*, *ATF3*, *FOSB*, *DUSP1*, and *TNFAIP3*) to perform analysis by qRT-PCR. Five genes, except UCP3, were significantly upregulated in T1D patients compared to the controls, similarly as we have found in the microarray experiments. *UCP3* expression was not significantly different between patients and controls (Figures 2(a)–2(f) and 3).

**Figure 4:**
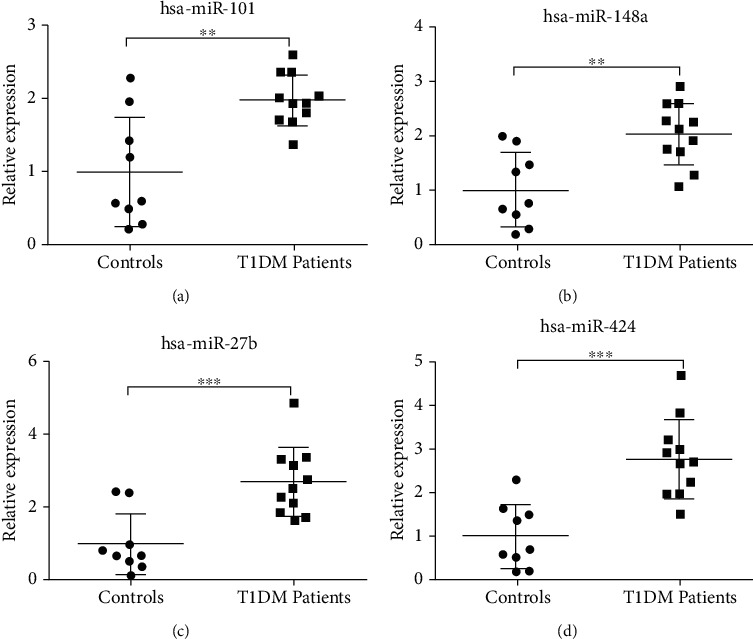
Relative expression of microRNAs evaluated by qRT-PCR, whose predicted targets are associated with oxidative stress response and DNA repair processes in PBMCs of T1D patients compared to the control group. The assay was performed for 11 samples of T1D patients and nine controls, which were the same used for the microarray method. Expression values of (a) *hsa-miR-101*, (b) *hsa-miR-148a*, (c) *hsa-miR-27b*, and (d) *hsa-miR-424* were normalized by the endogenous RNU48 gene. The bars represent the mean ± standard deviation. ∗∗ indicates statistically significant values for *p* < 0.01; ^∗∗∗^*p* < 0.001.

**Figure 5:**
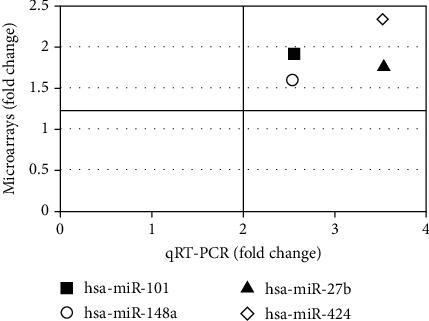
Comparison of fold change values (T1D vs. control group), obtained by the microarrays and qRT-PCR methods, for the selected differentially expressed microRNAs. The same samples (11 T1D patients and nine controls) were used for both methods.

Regarding the expression of microRNAs, we confirmed the microRNA expression (obtained in the microarray analysis) by performing the qRT-PCR for the following four microRNAs: *hsa-miR-101*, *hsa-miR148a*, *hsa-miR-27b*, and *hsa-miR-424*. All of them were significantly upregulated in T1D patients compared to the controls, compatible with the results obtained by the microarray method [21] (Figures 4(a)–4(d) and 5).

## 4. Discussion

The exact cause of T1D has not yet been elucidated, thus requiring a search for potential disease biomarkers and their possible functions related to the molecular mechanisms associated with the disease, providing new insights towards their application in clinical practice.

Among the 277 differentially expressed genes, it is important to note that we found some genes (*HLA-DQB1*, *CD69*, and *TNFAIP3*) that have been reported to be implicated in the pathogenesis of T1D [24]. While *HLA-DQB1* was downregulated, *CD69* and *TNFAIP3* were found upregulated. In addition, a series of genes highlighted in this study are involved in molecular and cellular events associated with T1D (*IL1B*, *TNF*, *IFNG*, *GZMB*, and *GZMH*) [25, 26] and inflammatory processes (*IL1B*, *IFNG*, *IL6*, *PTX3*, *CCL20*, *CXCL2*, and *DUSP2*) [3, 27], endorsing other studies in the literature. Interestingly, our results indicated differential expression of genes related to T1D even years after diagnosis, such as *CCL3L3*, *CCL4*, *CXCL1*, *CXCL3*, and *IL8 genes*.

In addition, the analysis by gene sets (GSA and GSEA) revealed an upregulation of biological processes related to ROS generation, oxidative stress, inflammation, cell death, ER stress, and DNA repair, among others. The excessive generation of reactive species (derived from oxygen or nitrogen) leads to a redox imbalance in the organism. Consequently, oxidative stress disturbs endoplasmic reticulum (ER) homeostasis and activates the unfolded protein response (UPR). Oxidative stress can also increase DNA damage, which may activate DNA repair and, depending on the scenario, may also lead to apoptosis [28, 29]. Other studies have described upregulation of DNA repair, inflammation, ER-stress response, and apoptosis pathways in T1D patients [30, 31]. There is also evidence that oxidative stress and inflammation occur even several months before the onset of T1D in children, favoring the autoimmunity and induced cellular damage in *β*-cells [32].

In this study, the high expression levels of *DUSP1*, *PTGS2*, *TNFAIP3*, *ATF3*, and *FOSB* genes in T1D compared with the control group were identified by the microarray analysis and confirmed by the qRT-PCR method. *DUSP1* (dual-specificity protein phosphatase-1), also known as MKP1, encodes a phosphatase protein that dephosphorylates and inactivates MAPKs (mitogen-activated protein kinases), playing an essential role in cell proliferation, cellular growth, inflammation, cell cycle arrest, innate immune function, and cellular response to oxidative damage [33]. In fact, it has been shown an upregulation of *DUSP1* under stress conditions that lead to apoptosis [34]. The gene *PTGS2* (prostaglandin synthase cyclooxygenase 2) encodes cyclooxygenase 2 (COX2) that generates prostaglandin E2 (PGE2). Stress conditions such as hyperglycemia and ROS induce the expression of *PTGS2* increasing PGE2 levels, which further stimulate ROS production [35, 36]. Elevated expression of *PTGS2* is commonly observed in many chronic inflammatory diseases, and high COX2 levels are also associated with microvascular complications of diabetes, including endothelial dysfunction and renal injuries [37].

*TNFAIP3* (tumor necrosis factor (TNF) *α*-induced protein-3) encodes a zinc finger protein (A20), which inhibits NFKB signaling, decreases NO production, and has demonstrated a critical role in protecting *β*-cells from apoptosis [38–40]. Interestingly, we found an upregulation of NFKB Inhibitor Alpha (*NFKBIA*) and NFKB Inhibitor Zeta (*NFKBIZ*) genes, both with inhibitory functions upon NFKB, which may be related to the high level of inflammation in T1D patients [41]. *TNFAIP3* has also been indicated as a candidate gene for T1D [42]. Some authors suggest that the SNP rs2327832 of *TNFAIP3* may help to predict glycemic control and disease progression in T1D children [39].

*ATF3* (activating transcription factor 3) gene is an adaptive-response gene activated upon various stress stimuli, including ROS, DNA damage, genotoxic agents, and cytokines[43]. There is evidence that *ATF3* silencing increases apoptosis in *β*-cells, thus suggesting a protective role regarding antiapoptotic effects [44]. ATF3 also represses inflammatory responses (by inhibiting NFKB), several proinflammatory cytokines, and NO synthesis [45].

Regarding *FOSB* (FosB Proto-Oncogene, AP-1 Transcription Factor Subunit), this gene encodes leucine zipper proteins that form dimers with other proteins (e.g., Jun and ATF/CREB) to compose the AP-1 complex which plays a crucial role in DNA repair mechanism [46]. Altogether, the upregulation of the aforementioned genes in T1D group may indicate that T1D patients exhibit an adaptive response activating cellular stress response and DNA repair genes upon several conditions, such as ROS, DNA damage, oxidative stress, and inflammation.

Moreover, microRNAs are important posttranscriptional regulators of several biological processes in T1D patients. Notably, it has been demonstrated that microRNAs show a specific signature for each form of diabetes (T1D, type 2 diabetes, or gestational diabetes) [47] and may be implicated in the pathogenesis and complications of diabetes [48]. Therefore, we crossed our differentially expressed mRNAs with the set of 44 differentially expressed microRNAs, which were previously reported for the same group of T1D patients [21]. Our results indicated that 52% of differentially expressed genes are predicted targets of the 44 microRNAs. Those target genes are related to inflammation, stress response, and DNA repair, showing the importance of microRNA regulation in these processes. In this study, we also confirmed the expression of four microRNAs (*hsa-miR-101*, *hsa-miR148a*, *hsa-miR-27b*, and *hsa-miR-424*) by qRT-PCR, all of them being upregulated in PBMCs of T1D patients.

miR-101 has been found to regulate DNA-dependent protein kinase (DNA-PKcs) and *ATM* (ataxia telangiectasia mutated) gene, with roles in suppressing DNA repair processes and sensitize cells to DNA damage induction [49]. Besides, there is evidence confirming that miR-101 targets *DUSP1*, promoting the activation of MAPKs and stimulating the production of proinflammatory cytokines [50]. On the other hand, miR-101 targets *PTGS2*, decreasing the release of proinflammatory cytokines [51]. miR-101 is also related to cytokine-mediated defective insulin production [52], *β*-cell dysfunction [52, 53], and increased apoptosis [52]. Interestingly, upregulation of miR-101 has been found in individuals with normal glucose levels, but testing positive for autoantibodies linked to T1D, suggesting that miR-101 precede the impairment of glucose homeostasis and may represent a potential biomarker for the onset of T1D [53]. In addition, miR-101 downregulation ameliorates insulin release and protects *β*-cells from cytokine-induced apoptosis [52].

Overexpression of miR-27b was found to inhibit NRF2 activation, a master regulator of antioxidant response [54], and suppress NFKB activation [55]. Furthermore, mir-27b upregulation has been associated with the onset of retinopathy [56], while its downregulation has been implicated in glucose tolerance impairment [57] and nephropathy [58]. The upregulation of mir-148a has been consistently found in T1D patients [59, 60]. miR-148a has been described as a regulator of autoimmunity, *β*-cell tolerance [18], and insulin activity [61]. Recently, Tamara et al. [62] reported that upregulation of miR-424 in T1D patients was associated with increased levels of inflammatory cytokines and increased risk of cardiovascular diseases.

Altogether, our results indicate specific mRNA and microRNA expression profiles in PBMCs from T1D patients relative to healthy nondiabetic individuals. In addition, we have shown that a number of biological processes related to oxidative stress response, DNA repair, inflammation, and apoptosis are upregulated in T1D patients. Besides, we provide new data regarding potential microRNA-mRNA interactions in T1D, in particular involving genes associated with responses to oxidative stress and DNA repair, which might play relevant roles in the microRNA-target network in T1D patients.

## Figures and Tables

**Table 1:** Main clinical characteristics of type 1 diabetes (T1D) patients.

Patient	Age (years)	Sex	Duration of T1D (years)	Insulin	Fasting glucose levels (mg/dL)	HbA1c (%)	HLA-DQB1
T1D_01	36	M	11	NPH 24 + 16 UI; regular 13 UI	213	10.8	∗0302 ∗0602
T1D_02^1^	23	M	13	Lanthus 28 UI; Lispro 4 + 2 + 2 UI	197	8.3	∗0301 ∗0302
T1D_03^1^	24	M	6	NPH 36 + 30 UI; regular 6 + 4 UI	260	10	∗0302 ∗0501
T1D_04^1^	18	M	8	NPH 28 + 24 UI; regular 10 + 10 UI	23	7.2	∗0201 ∗0501
T1D_05^2^	23	M	20	NPH 50 + 30 UI; regular 12 + 6 + 6 UI	178	10.1	∗0202 ∗0501
T1D_06^1^	21	F	8	Ultrafast 42 + 6 UI	223	7.8	∗0301 ∗0501
T1D_07^1^	29	M	2	NPH 20 + 12 UI; regular 6 UI	59	11.1	∗0301 ∗0301
T1D_08^1^	30	M	14	NPH 36 + 36 UI; regular 10 + 10 UI	47	8.9	∗0201 ∗0501
T1D_09^1^	21	M	16	NPH 54 + 10 UI; regular 8 + 10 UI	66	9	∗0301 ∗0501
T1D_10	28	F	5	NPH 34 + 22 UI	193	12.5	∗0501 ∗0605
T1D_11	29	M	3	NPH 24 + 12 UI; regular 4 UI	225	9.8	∗0302 ∗0604
T1D_12	27	F	10	NPH 24 + 12 UI; regular 6 + 6 UI	257	10.4	∗0201 ∗0302
T1D_13	37	F	7	Lanthus 44 UI; Aspart 4 + 5 + 2 UI	82	8.4	∗0501 ∗0501
T1D_14^1^	24	F	14	Lanthus 34 UI; regular 5 + 7 + 7 UI	293	8.5	∗0302 ∗0501
T1D_15	22	F	13	NPH 40 + 20 UI	143	8.3	∗0303 ∗0501
T1D_16^1^	18	M	5	NPH 20 + 10 + 15 UI; regular 10 + 8 + 8 UI	60	9.5	∗0301 ∗0302
T1D_17^1,2^	25	F	6	NPH 66+6 UI; regular 4 + 8 + 14 UI	85	10.5	∗0301 ∗0402
T1D_18	23	M	11	Glagina 34 UI; Aspart 4 + 6 + 6 UI	123	10.3	∗0301 ∗0602
T1D_19^1^	25	M	8	Levemir 20 + 20 UI	162	7.7	∗0301 ∗0501

^1^Patients whose samples were used in the microRNA expression study. It is noteworthy that all the 19 samples were used in the analysis of mRNA expression data. ^2^Patients using metformin (850 mg).

**Table 2:** Main characteristics of healthy individuals (control group).

Controls	Sex	Age (years)	Fasting glucose levels (mg/dL)	*HLA DQB1*
CO_01^1^	M	28	92	∗03:01 ∗03:02
CO_02^1^	F	25	86	∗02:01 ∗02:02
CO_03^1^	F	27	88	∗03:01 ∗03:02
CO_04^1^	F	26	90	∗04:02 ∗06:04
CO_05^1^	M	25	94	∗02:02 ∗03:01
CO_06^1^	F	29	82	∗02:02 ∗03:01
CO_07^1^	M	20	87	∗02:01 ∗06:03
CO_08^1^	F	20	87	∗03:01 ∗03:01
CO_09^1^	M	26	81	∗03:01 ∗03:03
CO_10	M	22	97	∗05:01 ∗06:03
CO_11	F	31	93	∗03:01 ∗03:02

^1^Controls whose samples were used for the study of microRNA expression profiles. All 11 samples were used in the analysis of mRNA expression data.

**Table 3:** List of differentially expressed genes, which have been widely related to T1D in the literature, in peripheral blood mononuclear cells (PBMCs) of T1D patients compared to the control group.

Gene	Gene name	Functions^1^	FC^2^	FDR^3^	*p*
*IL1B*	Interleukin 1, beta	Immune and inflammatory response, upregulation of T cell proliferation	2.60	0.00*E* + 00	0.00*E* + 00
*TNF*	Tumor necrosis factor	Humoral and inflammatory immune response, activation of MAPK activity	2.30	2.21*E* − 02	5.00*E* − 05
*CXCL2*	Chemokine (C-X-C motif) ligand 2	Inflammatory and immune response, chemotaxis	2.22	0.00*E* + 00	0.00*E* + 00
*CCL20*	Chemokine (C-C motif) ligand 20	Inflammatory and immune response, chemotaxis	1.70	7.00*E* − 04	0.00*E* + 00
*PTX3*	Pentraxin 3, long	Inflammatory response, phagocytosis and biosynthetic process of nitric oxide upregulation	1.62	0.00*E* + 00	0.00*E* + 00
*PTGS2*	Prostaglandin-endoperoxide synthase 2 (prostaglandin G/H synthase and cyclooxygenase)	Cell cycle regulation, response to cytokine stimulation and oxidative stress, biosynthetic process of prostaglandin	1.57	0.00*E* + 00	0.00*E* + 00
*TNFAIP3*	Tumor necrosis factor, alpha-induced protein 3	Antiapoptosis, negative regulation of NF-*κ*B transcription factor activity, TNF production, and inflammatory response	1.44	0.00*E* + 00	0.00*E* + 00
*ATF3*	Activating transcription factor 3	Transcription regulation	1.40	3.40*E* − 03	5.00*E* − 05
*DUSP2*	Dual specificity phosphatase 2	Inactivation of MAPK activity, regulation of the apoptotic process	1.38	0.00*E* + 00	0.00*E* + 00
*IL6*	Interleukin 6 (interferon, beta 2)	Humoral immune response, regulation of cell proliferation	1.37	4.00*E* − 04	0.00*E* + 00
*BCL2A1*	BCL2-related protein A1	Antiapoptosis	1.27	3.00*E* − 04	0.00*E* + 00
*GZMB*	Granzyme B (granzyme 2, cytotoxic T-lymphocyte-associated serine esterase 1)	Cytolysis, proteolysis, apoptotic process	1.22	5.40*E* − 03	0.00*E* + 00
*IFNG*	Interferon, gamma	Humoral and adaptive immune response, response to unfolded proteins of the endoplasmic reticulum, apoptotic process	1.17	2.87*E* − 02	3.00*E* − 04
*GZMH*	Granzyme H (cathepsin G-like 2, protein h-CCPX)	Cytolysis, proteolysis, apoptotic process	1.16	1.60*E* − 03	0.00*E* + 00
*CD69*	CD69 molecule	Lymphocyte proliferation, signal transmission receptor	1.14	3.16*E* − 02	4.00*E* − 04
*HLA-DQB1*	Major histocompatibility complex, class II, DQ beta 1	Immune response, upregulation of antigen processing and presentation	0.90	5.80*E* − 03	5.00*E* − 05

^1^The functions of all genes were obtained by the SOURCE tool (2000) (http://puma.princeton.edu/cgi-bin/source/sourceResult); ^2^FC: fold change; ^3^FDR: false discovery rate.

**Table 4:** Differentially expressed genes (with their respective fold-change values) obtained for PBMCs of T1D patients compared with the control group, highlighting genes belonging to the GO terms “response to oxidative stress” and “DNA repair,” according to the analysis performed by the Endeavor tool.

Rank	Oxidative stress response	Gene name	*Fold change*	DNA repair	Gene name	*Fold change*
**1**	*DUSP1*	*Dual specificity phosphatase 1*	1.43	*GADD45B*	*Growth arrest and DNA-damage-inducible, beta*	1.25
**2**	*UCP3*	*Uncoupling protein 3 (mitochondrial, proton carrier)*	0.81	*JUNB*	*Jun B protooncogene*	1.15
**3**	*PFKL*	*Phosphofructokinase, liver*	1.14	*PFKL*	*Phosphofructokinase, liver*	1.14
**4**	*PTGS2*	*Prostaglandin-endoperoxide synthase 2 (prostaglandin G/H synthase and cyclooxygenase)*	1.57	*ATF3*	*Activating transcription factor 3*	1.40
**5**	*PDGFRB*	*Platelet-derived growth factor receptor, beta polypeptide*	1.15	*FOSB*	*FBJ murine osteosarcoma viral oncogene homolog B*	1.43
**6**	*CCL4*	*Chemokine (C-C motif) ligand 4*	1.75	*PDGFRB*	*Platelet-derived growth factor receptor, beta polypeptide*	1.15
**7**	*CCR2*	*Chemokine (C-C motif) receptor 2*	0.80	*FOS*	*FBJ murine osteosarcoma viral oncogene homolog*	1.12
**8**	*LTF*	*Lactotransferrin*	0.97	*DDX3Y*	*DEAD (Asp-Glu-Ala-Asp) box helicase 3, Y-linked*	1.19
**9**	*CCL2*	*Chemokine (C-C motif) ligand 2*	1.18	*CDKN1C*	*Cyclin-dependent kinase inhibitor 1C (p57, Kip2)*	1.16
**10**	*MYOM2*	*Myomesin 2*	0.87	*PPP1R15A*	*Protein phosphatase 1, regulatory subunit 15A*	1.49
**11**	*LPL*	*Lipoprotein lipase*	1.31	*DUSP1*	*Dual specificity phosphatase 1*	1.43
**12**	*DSP*	*Desmoplakin*	1.10	*TNFAIP3*	*Tumor necrosis factor, alpha-induced protein 3*	1.44
**13**	*CES1*	*Carboxylesterase 1*	0.84	*MAPK8IP1*	*Mitogen-activated protein kinase 8 interacting protein 1*	0.89
**14**	*PPP1R15A*	*Protein phosphatase 1, regulatory subunit 15A*	1.49	*NFKBIA*	*Nuclear factor of kappa light polypeptide gene enhancer in B-cells inhibitor, alpha*	1.58
**15**	*TNFAIP3*	*Tumor necrosis factor, alpha-induced protein 3*	1.44	*LPL*	*Lipoprotein lipase*	1.31
**16**	*HP*	*Haptoglobin*	1.12	*DDX43*	*DEAD (Asp-Glu-Ala-Asp) box polypeptide 43*	1.09

**Table 5:** Potential microRNA-mRNA interactions selected from both lists of differentially expressed targets in PBMCs of T1D patients compared to controls.

Genes	MicroRNAs
*DUSP1*	hsa-let-7f; hsa-let-7g; hsa-miR-32; hsa-miR-98; hsa-miR-101; hsa-miR-148a; hsa-miR-148b
*UCP3*	hsa-let-7f; hsa-let-7g; hsa-miR-18b; hsa-miR-19a; hsa-miR-20b; hsa-miR-98; hsa-miR-148a; hsa-miR-148b; hsa-miR-454; hsa-miR-548c-3p; hsa-miR-301a; hsa-miR-324-5p
*PTGS2*	hsa-miR-7; hsa-miR-26b; hsa-miR-33a; hsa-miR-101; hsa-miR-148a; hsa-miR-148b; hsa-miR-542-3p; hsa-miR-548c-3p
*PDGFRB*	hsa-miR-18b; hsa-miR-19a; hsa-miR-20b; hsa-miR-338-3p; hsa-miR-342-3p; hsa-miR-423-5p; hsa-miR-766
*CCL4*	hsa-miR-542-3p
*CCL2*	hsa-miR-33a; hsa-miR-335
*LPL*	hsa-miR-27b; hsa-miR-32; hsa-miR-342-3p
*DSP*	hsa-miR-186; hsa-miR-548c-3p
*TNFAIP3*	hsa-let-7f; hsa-let-7g; hsa-miR-18b; hsa-miR-19a; hsa-miR-21; hsa-miR-27b; hsa-miR-98; hsa-miR-186; hsa-miR-548c-3p; hsa-miR-424; hsa-miR-423-5p
*GADD45B*	hsa-miR-454; hsa-miR-301a
*JUNB*	hsa-miR-101; hsa-miR-338-3p
*ATF3*	hsa-miR-7; hsa-miR-16; hsa-miR-27b; hsa-miR-195; hsa-miR-199a-3p; hsa-miR-542-3p; hsa-miR-424; hsa-miR-342-3p
*FOSB*	hsa-miR-27b; hsa-miR-148a; hsa-miR-148b; hsa-miR-338-3p; hsa-miR-548c-3p; hsa-miR-301a; hsa-miR-424; hsa-miR-342-3p; hsa-miR-342-5p; hsa-miR-423-5p; hsa-miR-766; hsa-miR-940
*FOS*	hsa-miR-7; hsa-miR-26b; hsa-miR-101; hsa-miR-199a-3p; hsa-miR-338-3p
*CDKN1C*	hsa-miR-32
*MAPK8IP1*	hsa-miR-10a; hsa-miR-338-3p; hsa-miR-542-3p; hsa-miR-940
*DDX43*	hsa-miR-548c-3p
*NFKBIA*	hsa-miR-140-3p

## Data Availability

The microarray data from all samples used in this study are publicly available in the ArrayExpress database under the accession numbers (https://www.ebi.ac.uk/arrayexpress/experiments/E-MEXP-3348 and https://www.ebi.ac.uk/arrayexpress/experiments/E-MEXP-3963).

## Supplementary Materials

Supplementary Materials Table S1: differentially expressed genes in PBMCs from T1D patients compared to control group obtained in mRNA microarray experiments. Table S2: differentially expressed/enriched biological processes in PBMCs from T1D patients compared to the control group obtained using BRB-ArrayTools.
