# Supplementary material for: Transcript Expression Profiles and MicroRNA Regulation Indicate an Upregulation of Processes Linked to Oxidative Stress, DNA Repair, Cell Death, and Inflammation in Type 1 Diabetes Mellitus Patients
Source: J Diabetes Res. 2022 Feb 1;2022:3511329. doi: 10.1155/2022/3511329 (PMC8825437; doi:10.1155/2022/3511329)
Supplement: Supplementary Materials — Table S1: differentially expressed genes in PBMCs from T1D patients compared to control group obtained in mRNA microarray experiments. Table S2: differentially expressed/enriched biological processes in PBMCs from T1D patients compared to the control group obtained using BRB-ArrayTools. [file 3511329.f1.docx]

**Supplementary Materials**

**Table S1.** Differentially expressed genes in peripheral blood mononuclear cells (PBMCs) from type 1 diabetes (T1D) patients compared to control group obtained in mRNA microarray experiments.

| **Genes** | ***Fold change*** | **FDR** | ***p-value*** |
| --- | --- | --- | --- |
| *A_24_P177553* | 1.2 | 3,34E-02 | 4,00E-04 |
| *A_24_P289043* | 1.19 | 4,61E-02 | 6,00E-04 |
| *A_24_P306469* | 1.19 | 2,74E-02 | 3,00E-04 |
| *A_24_P307306* | 1.16 | 1,74E-02 | 2,00E-04 |
| *A_24_P49597* | 0.87 | 1,30E-03 | 0,00E+00 |
| *A_24_P662366* | 1.26 | 2,80E-03 | 0,00E+00 |
| *A_24_P92267* | 1.15 | 3,68E-02 | 4,00E-04 |
| *ACBD4* | 1.26 | 0,00E+00 | 0,00E+00 |
| *ACBD5* | 1.22 | 1,46E-02 | 1,00E-04 |
| *ADM* | 1.2 | 2,80E-03 | 0,00E+00 |
| *ALAS2* | 1.13 | 0,00E+00 | 0,00E+00 |
| *ANXA3* | 1.15 | 4,90E-03 | 0,00E+00 |
| *APOBEC3A* | 1.22 | 7,00E-04 | 0,00E+00 |
| *APOBEC3B* | 1.13 | 3,66E-02 | 4,00E-04 |
| *APOL6* | 1.17 | 1,80E-03 | 0,00E+00 |
| *AQP9* | 1.09 | 2,12E-02 | 2,00E-04 |
| *AREG* | 1.42 | 0,00E+00 | 0,00E+00 |
| *ARL5B* | 1.15 | 4,29E-02 | 5,00E-04 |
| *ATF3* | 1.4 | 3,40E-03 | 5,00E-05 |
| *B3GAT1* | 1.22 | 0,00E+00 | 0,00E+00 |
| *BAMBI* | 1.19 | 2,85E-02 | 3,00E-04 |
| *BC137009* | 1.11 | 2,52E-02 | 3,00E-04 |
| *BCL2A1* | 1.27 | 3,00E-04 | 0,00E+00 |
| *BFSP1* | 1.18 | 3,15E-02 | 4,00E-04 |
| *C11orf96* | 1.26 | 1,20E-03 | 0,00E+00 |
| *C14orf181* | 1.22 | 1,79E-02 | 2,00E-04 |
| *C15orf38* | 0.88 | 2,70E-02 | 1,00E-04 |
| *C15orf48* | 1.32 | 0,00E+00 | 0,00E+00 |
| *C17orf87* | 1.08 | 0,00E+00 | 0,00E+00 |
| *C17orf91* | 1.16 | 1,92E-02 | 2,00E-04 |
| *C19orf33* | 0.84 | 2,72E-02 | 1,00E-04 |
| *C1orf229* | 1.01 | 4,02E-02 | 5,00E-04 |
| *C21orf7* | 1.29 | 1,50E-03 | 0,00E+00 |
| *C7orf16* | 1.35 | 0,00E+00 | 0,00E+00 |
| *CA6* | 1.18 | 2,28E-02 | 2,00E-04 |
| *CACNG6* | 0.83 | 1,03E-02 | 0,00E+00 |
| *CAMP* | 0.97 | 5,40E-03 | 0,00E+00 |
| *CCL2* | 1.18 | 3,69E-02 | 4,00E-04 |
| *CCL20* | 1.7 | 7,00E-04 | 0,00E+00 |
| *CCL3L3* | 2.69 | 1,34E-02 | 5,00E-05 |
| *CCL4* | 1.75 | 0,00E+00 | 0,00E+00 |
| *CCR2* | 0.8 | 4,57E-02 | 2,00E-04 |
| *CCRL2* | 1.29 | 5,00E-04 | 0,00E+00 |
| *CD69* | 1.14 | 3,16E-02 | 4,00E-04 |
| *CD83* | 1.75 | 0,00E+00 | 0,00E+00 |
| *CD8B* | 1.15 | 1,27E-02 | 1,00E-04 |
| *CDC42EP2* | 1.23 | 4,60E-03 | 0,00E+00 |
| *CDKN1C* | 1.16 | 2,36E-02 | 3,00E-04 |
| *CEACAM8* | 0.94 | 6,90E-03 | 5,00E-05 |
| *CENPK* | 1.19 | 1,50E-03 | 0,00E+00 |
| *CES1* | 0.84 | 1,50E-03 | 0,00E+00 |
| *CHI3L1* | 0.89 | 2,96E-02 | 1,00E-04 |
| *CISH* | 0.91 | 1,93E-02 | 2,00E-04 |
| *CLC* | 0.76 | 1,20E-03 | 0,00E+00 |
| *CLEC4G* | 0.92 | 2,90E-02 | 1,00E-04 |
| *CLEC4M* | 1.14 | 1,88E-02 | 2,00E-04 |
| *CNN1* | 1.05 | 1,42E-02 | 1,00E-04 |
| *CPA3* | 0.84 | 4,06E-02 | 1,00E-04 |
| *CSRNP1* | 1.24 | 1,24E-02 | 1,00E-04 |
| *CTSG* | 1.12 | 1,28E-02 | 5,00E-05 |
| *CXCL1* | 1.66 | 0,00E+00 | 0,00E+00 |
| *CXCL10* | 1.26 | 5,00E-04 | 0,00E+00 |
| *CXCL2* | 2.22 | 0,00E+00 | 0,00E+00 |
| *CXCL3* | 1.98 | 0,00E+00 | 0,00E+00 |
| *CYB5R2* | 1.05 | 4,71E-02 | 6,00E-04 |
| *CYorf15A* | 1.3 | 2,06E-02 | 5,00E-05 |
| *CYorf15B* | 1.22 | 2,05E-02 | 5,00E-05 |
| *DACT1* | 0.93 | 4,64E-02 | 2,00E-04 |
| *DDIT4* | 1.16 | 1,27E-02 | 1,00E-04 |
| *DDX3Y* | 1.19 | 6,00E-04 | 0,00E+00 |
| *DDX43* | 1.09 | 2,83E-02 | 3,00E-04 |
| *DEFA3* | 0.93 | 1,45E-03 | 0,00E+00 |
| *DEFA4* | 0.94 | 8,50E-04 | 0,00E+00 |
| *DEFB1* | 0.89 | 4,96E-02 | 2,00E-04 |
| *DIRC1* | 1.18 | 7,00E-04 | 0,00E+00 |
| *DOCK3* | 1.26 | 8,00E-04 | 0,00E+00 |
| *DPYSL4* | 0.87 | 4,12E-02 | 1,00E-04 |
| *DSP* | 1.1 | 0,00E+00 | 0,00E+00 |
| *DUSP1* | 1.43 | 0,00E+00 | 0,00E+00 |
| *DUSP2* | 1.38 | 0,00E+00 | 0,00E+00 |
| *EGR1* | 1.38 | 0,00E+00 | 0,00E+00 |
| *EGR2* | 1.19 | 1,55E-02 | 5,00E-05 |
| *EGR3* | 1.27 | 5,00E-04 | 0,00E+00 |
| *EIF1AY* | 1.32 | 0,00E+00 | 0,00E+00 |
| *EMP1* | 1.19 | 2,79E-02 | 3,00E-04 |
| *ENC1* | 1.28 | 7,10E-03 | 1,00E-04 |
| *ENST00000082259* | 1.18 | 3,08E-02 | 4,00E-04 |
| *ENST00000190839* | 1.05 | 4,50E-03 | 0,00E+00 |
| *ENST00000309874* | 1.06 | 2,06E-02 | 2,00E-04 |
| *ENST00000326989* | 1.17 | 1,18E-02 | 1,00E-04 |
| *ENST00000354689* | 1.2 | 1,58E-02 | 2,00E-04 |
| *ENST00000369123* | 0.9 | 2,26E-02 | 2,50E-04 |
| *ENST00000374057* | 1.26 | 5,00E-04 | 0,00E+00 |
| *ENST00000379350* | 1.13 | 7,00E-03 | 1,00E-04 |
| *ENST00000390539* | 1.27 | 0,00E+00 | 0,00E+00 |
| *ENST00000390549* | 1.27 | 5,00E-04 | 0,00E+00 |
| *ENST00000390625* | 1.3 | 3,60E-03 | 0,00E+00 |
| *ENST00000415386* | 1.04 | 2,00E-03 | 0,00E+00 |
| *ENST00000450856* | 1.38 | 7,00E-04 | 0,00E+00 |
| *ENST00000453166* | 1.22 | 7,10E-03 | 1,00E-04 |
| *ENST00000469435* | 1.19 | 1,75E-02 | 2,00E-04 |
| *ERAP1* | 1.12 | 2,23E-02 | 2,00E-04 |
| *ERAP2* | 1.11 | 2,30E-03 | 0,00E+00 |
| *EREG* | 1.35 | 0,00E+00 | 0,00E+00 |
| *FAM118A* | 0.87 | 2,53E-02 | 3,00E-04 |
| *FCGR1B* | 1.14 | 1,75E-02 | 2,00E-04 |
| *FCRL5* | 1.13 | 7,00E-04 | 0,00E+00 |
| *FFAR2* | 1.4 | 0,00E+00 | 0,00E+00 |
| *FGFBP2* | 1.25 | 7,00E-04 | 0,00E+00 |
| *FHL2* | 1.24 | 5,40E-03 | 0,00E+00 |
| *FOLR3* | 0.86 | 2,50E-03 | 0,00E+00 |
| *FOS* | 1.12 | 2,80E-03 | 0,00E+00 |
| *FOSB* | 1.43 | 0,00E+00 | 0,00E+00 |
| *FOXB1* | 1.25 | 3,00E-04 | 0,00E+00 |
| *G0S2* | 2.03 | 0,00E+00 | 0,00E+00 |
| *GADD45B* | 1.25 | 3,60E-03 | 0,00E+00 |
| *GBP1* | 1.15 | 3,09E-02 | 4,00E-04 |
| *GGNBP1* | 0.81 | 2,60E-03 | 0,00E+00 |
| *GOLGA6L6* | 1.31 | 0,00E+00 | 0,00E+00 |
| *GPR109B* | 1.42 | 0,00E+00 | 0,00E+00 |
| *GPR56* | 1.16 | 7,10E-03 | 1,00E-04 |
| *GPR84* | 1.13 | 1,13E-02 | 1,00E-04 |
| *GSTT1* | 1.01 | 1,23E-02 | 1,00E-04 |
| *GZMB* | 1.22 | 5,40E-03 | 0,00E+00 |
| *GZMH* | 1.16 | 1,60E-03 | 0,00E+00 |
| *HAGHL* | 1.17 | 7,00E-03 | 1,00E-04 |
| *HBA2* | 0.89 | 1,18E-02 | 1,00E-04 |
| *HBD* | 1.05 | 2,50E-03 | 0,00E+00 |
| *HBEGF* | 1.21 | 2,30E-03 | 0,00E+00 |
| *HBG1* | 1.36 | 2,04E-02 | 5,00E-05 |
| *HKDC1* | 0.82 | 4,92E-02 | 2,00E-04 |
| *HLA-DQA2* | 1.13 | 1,04E-02 | 0,00E+00 |
| *HLA-DQB1* | 0.9 | 5,80E-03 | 5,00E-05 |
| *HLA-DRB6* | 1.85 | 0,00E+00 | 0,00E+00 |
| *HP* | 1.12 | 5,00E-04 | 0,00E+00 |
| *HRASLS2* | 1.22 | 1,13E-02 | 1,00E-04 |
| *HSPB9* | 1.24 | 4,60E-03 | 0,00E+00 |
| *ICAM1* | 1.38 | 0,00E+00 | 0,00E+00 |
| *ID1* | 1.44 | 0,00E+00 | 0,00E+00 |
| *IER2* | 1.27 | 1,17E-02 | 1,00E-04 |
| *IER3* | 1.51 | 0,00E+00 | 0,00E+00 |
| *IFNG* | 1.17 | 2,87E-02 | 3,00E-04 |
| *IGJ* | 1.3 | 1,00E-03 | 0,00E+00 |
| *IL1A* | 1.49 | 8,50E-04 | 0,00E+00 |
| *IL1B* | 2.6 | 0,00E+00 | 0,00E+00 |
| *IL23A* | 1.19 | 1,20E-03 | 0,00E+00 |
| *IL4* | 0.82 | 1,08E-02 | 0,00E+00 |
| *IL6* | 1.37 | 4,00E-04 | 0,00E+00 |
| *IL8* | 1.99 | 0,00E+00 | 0,00E+00 |
| *ITGAD* | 1.14 | 6,10E-03 | 0,00E+00 |
| *JUNB* | 1.15 | 2,24E-02 | 2,00E-04 |
| *KANK1* | 0.92 | 4,16E-02 | 1,00E-04 |
| *KCNJ2* | 1.41 | 1,37E-02 | 5,00E-05 |
| *KDM5D* | 1.36 | 2,08E-02 | 5,00E-05 |
| *KIF19* | 1.3 | 0,00E+00 | 0,00E+00 |
| *KIR2DS2* | 1.06 | 3,42E-02 | 4,00E-04 |
| *KRT72* | 0.98 | 3,62E-02 | 4,00E-04 |
| *KRTAP1-3* | 1.16 | 1,70E-02 | 2,00E-04 |
| *LAIR2* | 0.98 | 2,15E-03 | 0,00E+00 |
| *LDLR* | 1.21 | 1,93E-02 | 2,00E-04 |
| *LGALS2* | 0.93 | 4,71E-02 | 2,00E-04 |
| *LILRB5* | 0.95 | 7,70E-03 | 1,00E-04 |
| *LOC100131138* | 1.17 | 0,00E+00 | 0,00E+00 |
| *LOC100170939* | 0.79 | 6,60E-03 | 0,00E+00 |
| *LOC100508964* | 1.1 | 2,05E-02 | 2,00E-04 |
| *LOC253039* | 1.15 | 1,17E-02 | 1,00E-04 |
| *LOC654433* | 1.04 | 2,16E-02 | 5,00E-05 |
| *LOC728715* | 1.01 | 4,02E-02 | 5,00E-04 |
| *LPL* | 1.31 | 1,10E-03 | 0,00E+00 |
| *LRRN3* | 0.8 | 1,00E-02 | 0,00E+00 |
| *LTF* | 0.97 | 6,55E-03 | 5,00E-05 |
| *MAB21L2* | 1.18 | 0,00E+00 | 0,00E+00 |
| *MAFB* | 1.16 | 2,87E-02 | 3,00E-04 |
| *MAFF* | 1.22 | 2,15E-02 | 2,00E-04 |
| *MAPK8IP1* | 0.89 | 6,10E-03 | 0,00E+00 |
| *MARCKS* | 1.27 | 2,00E-03 | 0,00E+00 |
| *MARCO* | 0.75 | 1,00E-03 | 0,00E+00 |
| *MDGA1* | 0.75 | 0,00E+00 | 0,00E+00 |
| *METRNL* | 1.26 | 1,60E-03 | 0,00E+00 |
| *MIAT* | 1.21 | 4,90E-03 | 0,00E+00 |
| *MME* | 0.91 | 4,54E-02 | 2,00E-04 |
| *MMP17* | 1.14 | 3,67E-02 | 4,00E-04 |
| *MOP-1* | 1.4 | 0,00E+00 | 0,00E+00 |
| *MT1M* | 1.22 | 1,31E-02 | 1,00E-04 |
| *MYL9* | 0.9 | 4,88E-02 | 2,00E-04 |
| *MYOM2* | 0.87 | 0,00E+00 | 0,00E+00 |
| *NCRNA00185* | 1.13 | 5,00E-04 | 0,00E+00 |
| *ND6* | 1.19 | 1,27E-02 | 1,00E-04 |
| *NEBL* | 1.05 | 2,15E-02 | 2,00E-04 |
| *NFKBIA* | 1.58 | 0,00E+00 | 0,00E+00 |
| *NFKBIZ* | 1.39 | 0,00E+00 | 0,00E+00 |
| *NGFRAP1* | 1.2 | 4,59E-02 | 6,00E-04 |
| *NKX1-2* | 1.17 | 7,70E-03 | 1,00E-04 |
| *NLRP3* | 1.07 | 4,40E-02 | 6,00E-04 |
| *NR4A2* | 1.3 | 7,00E-04 | 0,00E+00 |
| *NR4A3* | 1.19 | 2,22E-02 | 2,00E-04 |
| *NUAK1* | 1.07 | 1,89E-02 | 2,00E-04 |
| *OASL* | 1.17 | 1,23E-02 | 1,00E-04 |
| *OLFM4* | 1.13 | 1,15E-02 | 5,00E-05 |
| *OLIG1* | 0.82 | 1,62E-02 | 0,00E+00 |
| *OSM* | 1.1 | 2,80E-03 | 0,00E+00 |
| *PASK* | 1.15 | 2,07E-02 | 2,00E-04 |
| *PDGFRB* | 1.15 | 1,32E-02 | 1,00E-04 |
| *PDK4* | 0.82 | 4,65E-02 | 2,00E-04 |
| *PER1* | 1.17 | 2,29E-02 | 2,00E-04 |
| *PF4V1* | 1.35 | 0,00E+00 | 0,00E+00 |
| *PFKFB3* | 1.24 | 7,00E-04 | 0,00E+00 |
| *PFKL* | 1.14 | 3,77E-02 | 5,00E-04 |
| *PHLDA2* | 1.41 | 0,00E+00 | 0,00E+00 |
| *PIM3* | 1.26 | 8,60E-03 | 1,00E-04 |
| *PLA2G4C* | 1.17 | 8,70E-03 | 1,00E-04 |
| *PLAUR* | 1.12 | 3,14E-02 | 4,00E-04 |
| *PLEK* | 1.12 | 4,67E-02 | 6,00E-04 |
| *PLK2* | 1.27 | 6,70E-03 | 1,00E-04 |
| *PNMAL1* | 1.04 | 1,21E-02 | 1,00E-04 |
| *PPIF* | 1.2 | 2,76E-02 | 3,00E-04 |
| *PPP1R15A* | 1.49 | 0,00E+00 | 0,00E+00 |
| *PRF1* | 1.18 | 1,40E-02 | 1,00E-04 |
| *PRKY* | 1.13 | 4,10E-02 | 5,00E-04 |
| *PROK2* | 1.11 | 1,98E-02 | 2,00E-04 |
| *PRSS23* | 1.2 | 1,80E-03 | 0,00E+00 |
| *PRSS36* | 1.26 | 0,00E+00 | 0,00E+00 |
| *PRUNE2* | 0.9 | 4,58E-02 | 2,00E-04 |
| *PSPH* | 1.21 | 2,90E-03 | 0,00E+00 |
| *PTGS2* | 1.57 | 0,00E+00 | 0,00E+00 |
| *PTX3* | 1.62 | 0,00E+00 | 0,00E+00 |
| *PVRL2* | 1.07 | 3,71E-02 | 5,00E-04 |
| *PYY2* | 0.87 | 2,36E-02 | 1,00E-04 |
| *RAP1GAP2* | 1.18 | 1,50E-03 | 0,00E+00 |
| *RASIP1* | 1.33 | 8,00E-04 | 0,00E+00 |
| *RBPMS2* | 0.88 | 4,02E-02 | 1,00E-04 |
| *RCAN2* | 1.16 | 3,15E-02 | 4,00E-04 |
| *RGS1* | 1.16 | 4,90E-03 | 0,00E+00 |
| *RPS26* | 0.89 | 6,00E-03 | 0,00E+00 |
| *RPS4Y1* | 1.89 | 8,50E-04 | 0,00E+00 |
| *RPS4Y2* | 1.96 | 7,00E-04 | 0,00E+00 |
| *S100B* | 1.63 | 0,00E+00 | 0,00E+00 |
| *S1PR5* | 1.22 | 1,14E-02 | 1,00E-04 |
| *SCARF2* | 1.13 | 2,15E-02 | 2,00E-04 |
| *SELENBP1* | 1.15 | 0,00E+00 | 0,00E+00 |
| *SGK1* | 1.15 | 5,40E-03 | 0,00E+00 |
| *SHISA7* | 1.13 | 1,24E-02 | 1,00E-04 |
| *SIK1* | 1.26 | 1,20E-03 | 0,00E+00 |
| *SLC1A7* | 1.22 | 1,00E-03 | 0,00E+00 |
| *SLED1* | 1.29 | 0,00E+00 | 0,00E+00 |
| *SMAD1* | 1.22 | 2,87E-02 | 3,00E-04 |
| *SNAI1* | 1.24 | 7,00E-04 | 0,00E+00 |
| *SOCS3* | 1.33 | 0,00E+00 | 0,00E+00 |
| *SPOCD1* | 1.28 | 7,00E-04 | 0,00E+00 |
| *SPON2* | 1.15 | 3,56E-02 | 4,00E-04 |
| *SYNGR4* | 1.19 | 1,00E-03 | 0,00E+00 |
| *TACSTD2* | 0.76 | 0,00E+00 | 0,00E+00 |
| *TMEM176A* | 0.73 | 1,46E-02 | 1,50E-04 |
| *TMEM176B* | 0.81 | 2,70E-03 | 0,00E+00 |
| *TMEM95* | 0.77 | 4,83E-02 | 2,00E-04 |
| *TMTC1* | 0.94 | 4,90E-02 | 2,00E-04 |
| *TNF* | 2.3 | 2,21E-02 | 5,00E-05 |
| *TNFAIP3* | 1.44 | 0,00E+00 | 0,00E+00 |
| *TNFAIP6* | 1.33 | 1,00E-02 | 0,00E+00 |
| *TNFRSF17* | 1.33 | 4,00E-04 | 0,00E+00 |
| *TTTY15* | 1.21 | 0,00E+00 | 0,00E+00 |
| *TUBB2A* | 1.08 | 5,20E-03 | 0,00E+00 |
| *UCP3* | 0.81 | 9,70E-03 | 0,00E+00 |
| *UTS2* | 0.89 | 4,05E-03 | 0,00E+00 |
| *UTY* | 1.17 | 8,00E-04 | 0,00E+00 |
| *VASH1* | 0.83 | 4,97E-02 | 2,00E-04 |
| *VIT* | 1.2 | 1,46E-02 | 1,00E-04 |
| *VMO1* | 1.27 | 1,00E-03 | 0,00E+00 |
| *VSTM2L* | 0.84 | 1,80E-03 | 0,00E+00 |
| *WNT2B* | 0.85 | 2,71E-02 | 1,00E-04 |
| *XR_041758* | 1.25 | 7,00E-03 | 1,00E-04 |
| *XR_079018* | 1.13 | 4,24E-02 | 5,00E-04 |
| *ZFP36* | 1.33 | 0,00E+00 | 0,00E+00 |
| *ZFP57* | 0.88 | 9,10E-03 | 0,00E+00 |
| *ZFY* | 1.18 | 0,00E+00 | 0,00E+00 |
| *ZNF205* | 1.14 | 7,00E-03 | 1,00E-04 |
| *ZNF843* | 1.18 | 1,90E-03 | 0,00E+00 |

**Table S2.** Differentially expressed/enriched biological processes in PBMCs from T1D patients compared to the control group obtained using BRB-ArrayTools.

| GO | Category | GO term | Number of Genes | p-value of LS permutation | p-value of KS permutation | GSA Efron-Tibshirani test p-value |
| --- | --- | --- | --- | --- | --- | --- |
| **GO:0006475** | BP | *internal protein amino acid acetylation* | 97 | 0.00398 | 0.07538 | 0.186 (+) |
| **GO:0018394** | BP | *peptidyl-lysine acetylation* | 94 | 0.00278 | 0.08927 | 0.17 (+) |
| **GO:0018393** | BP | *internal peptidyl-lysine acetylation* | 92 | 0.00229 | 0.08697 | 0.173 (+) |
| **GO:0016573** | BP | *histone acetylation* | 90 | 0.00366 | 0.08448 | 0.161 (+) |
| **GO:0034976** | BP | *response to endoplasmic reticulum stress* | 88 | 0.00189 | 0.16241 | 0.029 (-) |
| **GO:0008017** | MF | *microtubule binding* | 82 | 0.00468 | 0.00201 | 0.009 (-) |
| **GO:0006984** | BP | *ER-nucleus signaling pathway* | 81 | 0.00193 | 0.00389 | 0.019 (+) |
| **GO:0035967** | BP | *cellular response to topologically incorrect protein* | 76 | 0.00144 | 0.00218 | 0.023 (+) |
| **GO:0030968** | BP | *endoplasmic reticulum unfolded protein response* | 72 | 0.00236 | 0.00818 | 0.026 (+) |
| **GO:0034620** | BP | *cellular response to unfolded protein* | 72 | 0.00236 | 0.00818 | 0.026 (+) |
| **GO:0051213** | MF | *dioxygenase activity* | 70 | 0.42262 | 0.00182 | 0.303 (-) |
| **GO:0016701** | MF | *oxidoreductase activity. acting on single donors with incorporation of molecular oxygen* | 69 | 0.53757 | 0.00217 | 0.389 (-) |
| **GO:0097190** | BP | *apoptotic signaling pathway* | 68 | 0.00727 | 0.01973 | < 0.001 (+) |
| **GO:0022626** | CC | *cytosolic ribosome* | 68 | 0.0066 | 0.00384 | 0.038 (-) |
| **GO:0016702** | MF | *oxidoreductase activity. acting on single donors with incorporation of molecular oxygen. incorporation of two atoms of oxygen* | 68 | 0.51001 | 0.00261 | 0.362 (-) |
| **GO:0000123** | CC | *histone acetyltransferase complex* | 64 | 0.00099 | 0.01775 | 0.145 (+) |
| **GO:0032069** | BP | *regulation of nuclease activity* | 61 | 0.00395 | 0.03411 | 0.01 (+) |
| **GO:0032075** | BP | *positive regulation of nuclease activity* | 56 | 0.00183 | 0.00155 | 0.001 (+) |
| **GO:0051341** | BP | *regulation of oxidoreductase activity* | 52 | 0.06218 | 0.02343 | < 0.001 (+) |
| **GO:0042379** | MF | *chemokine receptor binding* | 49 | 0.00348 | 0.03645 | 0.004 (+) |
| **GO:0007033** | BP | *vacuole organization* | 49 | 0.00283 | 0.00117 | 0.032 (+) |
| **GO:0046209** | BP | *nitric oxide metabolic process* | 44 | 0.06804 | 0.00167 | 0.021 (+) |
| **GO:0000724** | BP | *double-strand break repair via homologous recombination* | 42 | 0.47862 | 0.00475 | 0.238 (+) |
| **GO:0000725** | BP | *recombinational repair* | 42 | 0.47862 | 0.00475 | 0.238 (+) |
| **GO:0004402** | MF | *histone acetyltransferase activity* | 39 | 0.00378 | 0.03139 | 0.053 (-) |
| **GO:0001085** | MF | *RNA polymerase II transcription factor binding* | 39 | 0.02052 | 0.18111 | 0.001 (-) |
| **GO:0032623** | BP | *interleukin-2 production* | 38 | 0.00182 | 0.00004 | 0.001 (+) |
| **GO:0045833** | BP | *negative regulation of lipid metabolic process* | 38 | 0.00451 | 0.08443 | 0.002 (+) |
| **GO:0006809** | BP | *nitric oxide biosynthetic process* | 38 | 0.06784 | 0.00391 | 0.026 (+) |
| **GO:0031047** | BP | *gene silencing by RNA* | 37 | 0.03785 | 0.00396 | 0.162 (-) |
| **GO:0001836** | BP | *release of cytochrome c from mitochondria* | 35 | 0.01415 | 0.09448 | 0.004 (+) |
| **GO:0032663** | BP | *regulation of interleukin-2 production* | 34 | 0.00222 | 0.0005 | < 0.001 (+) |
| **GO:0033613** | MF | *activating transcription factor binding* | 34 | 0.03362 | 0.11103 | < 0.001 (-) |
| **GO:0003678** | MF | *DNA helicase activity* | 33 | 0.00423 | 0.00151 | 0.131 (+) |
| **GO:0000149** | MF | *SNARE binding* | 33 | 0.19052 | 0.42141 | 0.004 (-) |
| **GO:0070588** | BP | *calcium ion transmembrane transport* | 32 | 0.05266 | 0.53295 | 0.003 (-) |
| **GO:0043331** | BP | *response to dsRNA* | 31 | 0.07055 | 0.11695 | 0.002 (+) |
| **GO:1901216** | BP | *positive regulation of neuron death* | 28 | 0.00894 | 0.13239 | < 0.001 (+) |
| **GO:0045576** | BP | *mast cell activation* | 28 | 0.25033 | 0.00439 | 0.04 (-) |
| **GO:0006206** | BP | *pyrimidine nucleobase metabolic process* | 27 | 0.08276 | 0.00351 | 0.114 (-) |
| **GO:0005637** | CC | *nuclear inner membrane* | 26 | 0.09972 | 0.39367 | 0.002 (+) |
| **GO:0019905** | MF | *syntaxin binding* | 26 | 0.19875 | 0.3242 | 0.004 (-) |
| **GO:0009409** | BP | *response to cold* | 25 | 0.11662 | 0.17817 | 0.002 (+) |
| **GO:0051055** | BP | *negative regulation of lipid biosynthetic process* | 25 | 0.00451 | 0.02373 | 0.011 (+) |
| **GO:0032392** | BP | *DNA geometric change* | 25 | 0.00389 | 0.0005 | 0.125 (+) |
| **GO:0090199** | BP | *regulation of release of cytochrome c from mitochondria* | 24 | 0.00596 | 0.0239 | 0.003 (+) |
| **GO:0043044** | BP | *ATP-dependent chromatin remodeling* | 24 | 0.04205 | 0.00427 | 0.146 (-) |
| **GO:0032570** | BP | *response to progesterone stimulus* | 22 | 0.09085 | 0.00291 | 0.007 (+) |
| **GO:0032508** | BP | *DNA duplex unwinding* | 22 | 0.00214 | 0.00005 | 0.061 (+) |
| **GO:0071359** | BP | *cellular response to dsRNA* | 21 | 0.12548 | 0.37257 | 0.002 (+) |
| **GO:0042094** | BP | *interleukin-2 biosynthetic process* | 19 | 0.07463 | 0.00451 | 0.021 (+) |
| **GO:0035195** | BP | *gene silencing by miRNA* | 19 | 0.0596 | 0.00428 | 0.133 (+) |
| **GO:0010677** | BP | *negative regulation of cellular carbohydrate metabolic process* | 16 | 0.01814 | 0.05572 | 0.002 (+) |
| **GO:0005048** | MF | *signal sequence binding* | 15 | 0.00257 | 0.01827 | 0.001 (+) |
| **GO:0044253** | BP | *positive regulation of multicellular organismal metabolic process* | 15 | 0.03454 | 0.40034 | < 0.001 (-) |
| **GO:0002026** | BP | *regulation of the force of heart contraction* | 15 | 0.14803 | 0.12805 | < 0.001 (+) |
| **GO:0080008** | CC | *Cul4-RING ubiquitin ligase complex* | 15 | 0.23394 | 0.00295 | 0.294 (-) |
| **GO:0017069** | MF | *snRNA binding* | 14 | 0.01641 | 0.00321 | 0.098 (+) |
| **GO:0033549** | MF | *MAP kinase phosphatase activity* | 14 | 0.03794 | 0.13552 | 0.001 (+) |
| **GO:0072215** | BP | *regulation of metanephros development* | 14 | 0.02606 | 0.00028 | 0.065 (+) |
| **GO:0048820** | BP | *hair follicle maturation* | 13 | 0.02726 | 0.02152 | < 0.001 (+) |
| **GO:0015923** | MF | *mannosidase activity* | 11 | 0.00173 | 0.00949 | 0.012 (-) |
| **GO:0008171** | MF | *O-methyltransferase activity* | 11 | 0.03896 | 0.00164 | 0.001 (+) |
| **GO:0016862** | MF | *intramolecular oxidoreductase activity. interconverting keto- and enol-groups* | 11 | 0.04342 | 0.00228 | 0.088 (-) |
| **GO:0004559** | MF | *alpha-mannosidase activity* | 10 | 0.00207 | 0.00781 | 0.021 (-) |
| **GO:0031063** | BP | *regulation of histone deacetylation* | 10 | 0.06998 | 0.23074 | 0.002 (-) |
| **GO:0007253** | BP | *cytoplasmic sequestering of NF-kappaB* | 10 | 0.0659 | 0.30501 | 0.004 (+) |
| **GO:0090312** | BP | *positive regulation of protein deacetylation* | 10 | 0.01836 | 0.01913 | 0.003 (-) |
| **GO:0005149** | MF | *interleukin-1 receptor binding* | 10 | 0.07031 | 0.00399 | 0.001 (+) |
| **GO:0016471** | CC | *vacuolar proton-transporting V-type ATPase complex* | 10 | 0.08149 | 0.31101 | 0.003 (-) |
| **GO:0030276** | MF | *clathrin binding* | 8 | 0.0183 | 0.0138 | 0.004 (-) |
| **GO:0071545** | BP | *inositol phosphate catabolic process* | 8 | 0.06538 | 0.169 | 0.001 (-) |
| **GO:0001562** | BP | *response to protozoan* | 8 | 0.0367 | 0.05555 | 0.002 (+) |
| **GO:0018342** | BP | *protein prenylation* | 8 | 0.12377 | 0.13394 | 0.004 (-) |
| **GO:0097354** | BP | *prenylation* | 8 | 0.12377 | 0.13394 | 0.004 (-) |
| **GO:0016722** | MF | *oxidoreductase activity. oxidizing metal ions* | 8 | 0.12651 | 0.00064 | 0.027 (-) |
| **GO:0015924** | MF | *mannosyl-oligosaccharide mannosidase activity* | 7 | 0.00156 | 0.00115 | 0.012 (-) |
| **GO:0046838** | BP | *phosphorylated carbohydrate dephosphorylation* | 7 | 0.04541 | 0.06769 | 0.001 (-) |
| **GO:0009185** | BP | *ribonucleoside diphosphate metabolic process* | 7 | 0.08313 | 0.10214 | 0.001 (-) |
| **GO:0030033** | BP | *microvillus assembly* | 7 | 0.06997 | 0.02769 | 0.004 (-) |
| **GO:0006515** | BP | *misfolded or incompletely synthesized protein catabolic process* | 7 | 0.02454 | 0.01847 | 0.004 (-) |
| **GO:0033194** | BP | *response to hydroperoxide* | 7 | 0.11274 | 0.00145 | 0.071 (-) |
| **GO:0070775** | CC | *H3 histone acetyltransferase complex* | 6 | 0.00366 | 0.05757 | 0.088 (+) |
| **GO:0070567** | MF | *cytidylyltransferase activity* | 6 | 0.02648 | 0.0001 | 0.013 (+) |
| **GO:0032506** | BP | *cytokinetic process* | 6 | 0.0149 | 0.00078 | < 0.001 (-) |
| **GO:0044380** | BP | *protein localization to cytoskeleton* | 6 | 0.0033 | 0.00045 | 0.006 (-) |
| **GO:0019908** | CC | *nuclear cyclin-dependent protein kinase holoenzyme complex* | 5 | 0.01192 | 0.05898 | 0.002 (-) |
| **GO:0010340** | MF | *carboxyl-O-methyltransferase activity* | 5 | 0.03523 | 0.05061 | < 0.001 (+) |
| **GO:0051998** | MF | *protein carboxyl O-methyltransferase activity* | 5 | 0.03523 | 0.05061 | < 0.001 (+) |
| **GO:2001026** | BP | *regulation of endothelial cell chemotaxis* | 5 | 0.20382 | 0.17999 | 0.004 (+) |
| **GO:0072393** | BP | *microtubule anchoring at microtubule organizing center* | 5 | 0.00553 | 0.03282 | < 0.001 (-) |
| **GO:0009135** | BP | *purine nucleoside diphosphate metabolic process* | 5 | 0.03486 | 0.04226 | 0.002 (-) |
| **GO:0009179** | BP | *purine ribonucleoside diphosphate metabolic process* | 5 | 0.03486 | 0.04226 | 0.002 (-) |
| **GO:0003339** | BP | *regulation of mesenchymal to epithelial transition involved in metanephros morphogenesis* | 5 | 0.08499 | 0.00089 | 0.027 (+) |
| **GO:0046031** | BP | *ADP metabolic process* | 5 | 0.03486 | 0.04226 | 0.002 (-) |

BP – biological process; MF – molecular function; CC – cellular component.
